# Non-invasive assessment of coronary artery bypass graft patency using 16-slice computed tomography angiography

**DOI:** 10.1186/1749-8090-2-27

**Published:** 2007-06-05

**Authors:** Emma S Houslay, Tristan Lawton, Anshuman Sengupta, Neal G Uren, Graham McKillop, David E Newby

**Affiliations:** 1 Centre for Cardiovascular Science, University of Edinburgh, Edinburgh, UK; 2 Dept. of Radiology, University of Edinburgh, Edinburgh, UK; 3 Dept. of Cardiology, University of Edinburgh, Edinburgh, UK

## Abstract

**Background:**

Invasive coronary angiography is the gold standard means of imaging bypass vessels and carries a small but potentially serious risk of local vascular complications, including myocardial infarction, stroke and death. We evaluated computed tomography as a non-invasive means of assessing graft patency.

**Methods:**

Fifty patients with previous coronary artery bypass surgery who were listed for diagnostic coronary angiography underwent contrast enhanced computed tomography angiography using a 16-slice computed tomography scanner. Images were retrospectively gated to the electrocardiogram and two dimensional axial, multiplanar and three dimensional reconstructions acquired. Sensitivity, specificity, positive and negative predictive value, accuracy and level of agreement for detection of graft patency by multidetector computed tomography.

**Results:**

A total of 116 grafts were suitable for analysis. The specificity of CT for the detection of graft patency was 100%, with a sensitivity of 92.8%, positive predictive value 100%, negative predictive value 85.8% and an accuracy of 94.8%. The kappa value of agreement between the two means of measuring graft patency was 0.9. Mean radiation dose was 9.0 ± 7.2 mSv for coronary angiography and 18.5 ± 4 mSv for computed tomography. Pooled analysis of eight studies, incorporating 932 grafts, confirmed a 97% accuracy for the detection of graft patency by multidetector computed tomography.

**Conclusion:**

Computed tomography is an accurate, rapid and non-invasive method of assessing coronary artery bypass graft patency. However, this was achieved at the expense of an increase in radiation dose.

## Background

Coronary artery bypass grafting (CABG) was first performed in 1967 by Garrett et al[1], who successfully employed a saphenous vein graft (SVG) for the treatment of coronary artery disease. This procedure has now become a widespread treatment for intractable angina and, in high risk patients, improves survival[2]. However, the benefits of surgery may be lost with graft failure or occlusion. Vein graft patency has been found to be reduced to 81% at one year, 75% at 5 years and less than 50% at 15 years[3]. This has led to the increasing use of arterial conduits, such as left internal mammary grafts (LIMA), that are associated with improved long term (10–15 year) patency and survival[4,5].

Vein graft occlusion may occur early or late and is due to three distinct, well described disease processes. Acute graft failure and thrombosis may occur in the first 30 days postoperatively, and affects up to 12% of vein grafts[3]. Neointimal hyperplasia occurs between one month and a year, and is the result of accumulation of smooth muscle cells and extracellular matrix in the intimal compartment. While this rarely causes clinically significant stenosis[6], it provides the foundation for the development of graft atheroma. Late graft failure results from an accelerated form of atherosclerosis called 'graft vasculopathy'. This process predominates beyond the first year after surgery and is present in 17% of grafts at 6 years and 46% of grafts at 11 years[7].

The gold standard method of assessing graft patency is coronary angiography. This invasive procedure carries the small risk (10 in 1000) of potentially serious local vascular complications, including myocardial infarction, stroke and death. Studies have shown that elective coronary angiography in clinically stable patients with saphenous vein grafts carries a 0.08% risk of myocardial infarction, while 0.7% of subjects experienced clinically important complications. The risk of myocardial infarction increased to 1.3% for urgent studies[8]. The assessment of graft patency in a non-invasive readily applicable manner would have major benefits for the management and treatment of patients with prior CABG. The large calibre and more static location of bypass grafts make them particularly suitable for investigation by potential non-invasive imaging modalities. Computed tomography angiography was first described as a means of determining bypass graft patency in 1980[9,10]. With advances in spiral and multidetector computed tomography (MDCT) technology, there has emerged a growing body of evidence to support the use of computed tomography for non-invasive bypass graft assessment [11-20]. Using the reference gold-standard of invasive coronary angiography, we aimed to assess whether contrast enhanced MDCT can reliably predict graft patency in patients who have previously undergone CABG.

## Methods

### Patient population

Fifty consecutive patients who had undergone previous coronary artery bypass graft surgery and were listed for diagnostic coronary angiography between June 2004 and June 2005 were recruited into the study. Exclusion criteria were the presence of implanted metallic cardiac devices which may interfere with image quality (prosthetic heart valves, implantable pacemaker or cardiodefibrillator), renal impairment, atrial fibrillation or those patients unable to tolerate the supine position. The study was conducted with the approval of the local research ethics committee, in accordance with the Declaration of Helsinki and the written informed consent of each subject.

### Coronary angiogram

Invasive coronary angiography was performed by an experienced cardiologist via standard percutaneous approach, using 6 French Judkins catheters. Images were obtained on a Innova digital flat plate system (Advantx, GE Medical Systems) following i.v. bolus injection of iopamidol contrast agent (Niopam, Bracco, Bucks, UK). Selective catheterisation of grafts or graft stumps was performed.

### MDCT angiogram

MDCT angiography was performed using a 16-slice MDCT scanner (Aquilon; Toshiba, Tustin, CA). The scan volume was defined based on expected location of the coronary arteries and grafts, following a scout view. In patients with known internal mammary artery grafts, this area was extended to the origin of the IMA at the proximal subclavian arteries. Scanning parameter included a gantry rotation time of 0.5 seconds, 16 × 1 mm detector collimation, 0.35 to 0.5 × 0.35 to 1 mm voxel size resulting in 0.35 to 0.5 × 0.35 to 0.5 mm display voxel size on MPR workstation, 135 kV, 250 to 300 mA, 0.25 pitch, and inspiratory breath hold time 20–30 seconds. Iomeprol, 100 mL (400-strength, Bracco, Bucks, UK) contrast agent was administered intravenously at a flow rate of 3.5 mL/s. The ^SURE^Cardio acquisition feature was utilised, which monitors the patient's heart rate for five consecutive beats, calculates an average and automatically selects optimal scan parameters. The contrast bolus was monitored using the ^SURE^Start feature to initiate imaging when contrast density in the ascending aorta is 160–180 HU. The images were obtained during inspiratory breath hold and retrospectively cardiac gated.

**Figure 1:**
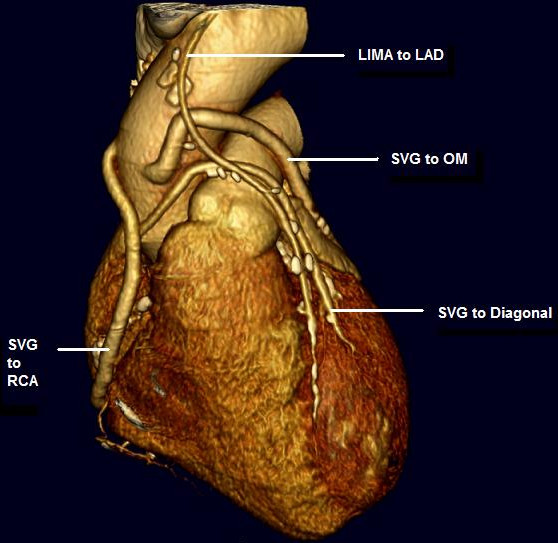
Three dimensional reconstruction with volume rendering techniques demonstrating saphenous vein grafts to the first diagonal branch of the left anterior descending artery, obtuse marginal artery and right coronary artery and a left internal mammary graft to the left anterior descending artery.

The reconstruction set was limited to one phase at 75% of the R-R interval. From these images, one slice was selected to demonstrate best the three main coronary arteries at the mid-heart level. The selected slice was then reconstructed for the entire cardiac cycle at 20 ms intervals. From these images, the phase which best demonstrated the coronary arteries at this slice position were selected and the entire volume reconstructed at the selected phase (Figure 1). The images were transferred to a dedicated workstation (Vitrea v3.5; Vital Images, Plymouth, MN).

### Data analysis

Two-dimensional axial, multiplanar reconstruction and three-dimensional reconstructions with volume rendering techniques were constructed (Voxar 3D, Voxar) and analysed by a radiologist who was familiar with the cardiac anatomy but blinded to the result of the invasive coronary angiogram. Image quality was graded in terms of eligible or insufficient (motion artefact, artefact caused by surgical clip) and eligible grafts were assessed in terms of patency. The results of MDCT were compared with invasive coronary angiography, the gold standard reference. Sensitivity, specificity, positive and negative predictive value (PPV and NPV respectively) was calculated for the detection of graft *patency *by MDCT. Sensitivity was calculated as true patent/(true patent + false occluded) grafts. Specificity was calculated as the number of true occluded/(true occluded + false patent) grafts. The positive predictive value was a result of true patent/(true patent + false patent), and the negative predictive value as true occluded/(true occluded + false occluded). The accuracy was determined by (true patent + true occluded)/total number of grafts. In addition, accuracy for detection of graft patency was calculated on a per patient basis.

Radiation dose for invasive coronary angiography and MDCT angiography was calculated from the documented dose area product (cGycm^2^) and dose length product (mGycm) respectively.

## Results

Patients were predominantly male, with a mean graft age of 7 ± 5 years (Table 1) and the majority (66%) were on beta-blockers. Mean heart rate was 67 beats per minute (range 52–89). MDCT angiography was performed on all 50 patients a mean of 55 days following invasive coronary angiography (range 40–74). Of these patients, two studies were not of sufficient diagnostic quality for image reconstruction due to arrhythmia and technical failure, thereby excluding 6 grafts from analysis. Image reconstruction was not possible on a further six grafts due to image artefact and a further one excluded due to stent insertion with subsequent in-stent stenosis between recruitment to the study and time of MDCT. Of 129 grafts, a total of 116 were suitable for image reconstruction and analysis. There were no complications as a result of the MDCT or invasive coronary angiography.

**Table 1:** Baseline Characteristics

**No. of patients**	**50**
**Sex (% male)**	**86**
**Mean age (SD)**	**66 (9) years**
**Heart Rate (Mean, Median Range)**	**67, 68, 52–89**
**No. of bypass grafts**	**116**
**No. of IMA grafts**	**35**
• IMA → LAD	30
• IMA → OM	2
• IMA → Diagonal	2
• IMA → Circumflex	1
**No. of SVGs**	**77**
• SVG → OM	26
• SVG → RCA	25
• SVG → LAD	13
• SVG → Diagonal	7
• SVG → posterior descending	6
**Radial artery grafts**	**4**
• → OM	3
• → posterior descending	1
**Mean graft age (SD)**	**7 (5) years**

IMA: internal mammary artery; SVG: saphenous vein graft; LAD: left anterior descending artery; OM: obtuse marginal artery; RCA: right coronary artery

**Figure 2:**
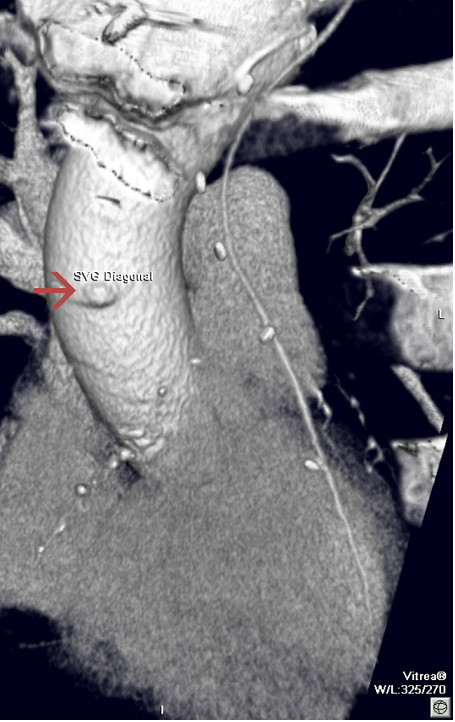
Occluded saphenous vein graft; only graft stump visible (arrow).

MDCT correctly identified 77 of 83 patent grafts and 33 of 33 occluded grafts (Table 2, Figs. 1 and 2). The sensitivity for detection of graft patency was 92.8%, specificity 100%, positive predictive value 100%, negative predictive value 84.6% and accuracy 94.8%. The main discrepancies lie in the reporting of IMA grafts, where there were a significant proportion of false occlusions (Table 3). There was a very good strength of agreement between the two imaging modalities (Cohen's κ = 0.9).

**Table 2:** Graft characteristics

	**Angiography**		**MDCT**			
	**Patent**	**Occluded**	**True patent**	**False patent**	**True occluded**	**False occluded**

**Overall n = 116**	83	33	77	0	33	6
**IMA n = 35**	30	5	25	0	5	5
**SVG n = 77**	52	25	51	0	25	1
**Other n = 4**	1	3	1	0	3	0

IMA: internal mammary artery; SVG: saphenous vein graft.

**Table 3:** Diagnostic accuracy of MDCT for detection of graft patency

	**Sensitivity (%)**	**Specificity (%)**	**PPV (%)**	**NPV (%)**	**Accuracy (%)**
**Overall n = 116**	**92.8**	**100**	**100**	**84.6**	**94.8**
**IMA grafts n = 35**	82.8	100	100	50	85.3
**SVG n = 77**	98.1	100	100	96.3	98.7
**Other n = 4**	100	100	100	100	100

IMA: internal mammary artery; SVG: saphenous vein graft; PPV: positive predictive value; NPV: negative predictive value.

### Radiation exposure

The mean radiation exposure for invasive coronary angiography was 9.0 ± 7.2 mSv with mean screening time of 11 ± 7.3 minutes. The mean radiation exposure for MDCT angiography was 18.5 ± 4 mSv with a mean scan time of 24 s (range 18–26).

## Discussion

Invasive coronary angiography of coronary artery bypass grafts, in particular selective catheterisation of arterial conduits, is technically demanding, time consuming and involves day case admission. MDCT provides a highly specific means of detecting coronary artery bypass graft patency in a clinical setting. It is safe and abolishes the need for invasive coronary angiography which is associated with the small but significant risk of major complications. In contrast to invasive coronary angiography, it can be performed on a scheduled out-patient basis rather than requiring admission to a day case unit.

Computed tomography was first investigated as a possible method of determining patency of coronary artery bypass grafts in the early 1980s. Gunthaner et al[10] found that they were able to determine patency in 77.5% of left anterior descending (LAD) and right coronary artery (RCA) grafts and 40% of obtuse marginal (OM) grafts. Brundage et al[9] also found 95% correlation between single slice CT and conventional angiographic detection of graft patency. With the advent of spiral and MDCT scanners, image quality has improved and, in line with this, interest in minimally invasive imaging techniques has grown. Since these first studies, further larger scale studies have been undertaken to assess the ability of CT to assess graft patency. Recently, Song and colleagues reported that MDCT imaging resulted in 99.4% specificity and 100% sensitivity, with 100% PPV and 80% NPV for detection of bypass graft patency[20]. This study, however, looked at patients (n=50) immediately post-coronary artery bypass operation, who may be expected to have a high patency rate and no graft vasculopathy, thereby minimising any ambiguity caused by poor flow in chronically diseased grafts. Recent studies (n = 25–65) looking specifically at graft patency in study populations similar to the present, have reported sensitivities of 90–98% and specificities of 88–100% for detection of graft patency[11,15,21]. Consistent with our findings, detection of patency in vein grafts is more reliable than for internal mammary grafts[12,22].

We performed a pooled analysis of all available data from studies investigating CT as a non-invasive means of assessing coronary artery bypass grafts (Table 4)[11,12,14,15,18,23-27]. The paper by Nieman *et al *was not included in the analysis due to incomplete data on detection of graft occlusion by MDCT[28]. The pooled data gave an overall sensitivity of 96% and specificity of 99% (n=1498) for the detection of graft patency. As we have observed, studies with a preponderance of vein grafts displayed more reliable patency rates than those with a large proportion of arterial grafts. Pooled analysis focussing on the breakdown of available data for IMAs (n = 268) gave a sensitivity of 93%, specificity 97%, PPV 99.5%, NPV 67% and an accuracy of 94%[11,12,14,15,18]. Pooled analysis of vein grafts in the same studies (n = 399) gave a sensitivity of 97%, specificity 97%, PPV 99%, NPV 92% and an accuracy of 97%.

Computed tomography has many advantages over invasive coronary angiography, including a lower complication rate, better ostial imaging and easy visualisation of vessels with anomalous origin and those where catheterisation has failed. MDCT in particular has the advantage of shorter breath hold times, faster gantry rotation and reduced slice thickness permitting better temporal and spatial resolution than previous CT scanners. MDCT is widely available in standard Radiology departments, unlike electron beam CT.

**Table 4:** Characteristics of studies assessing non-invasive imaging of coronary artery bypass grafts.

	Achenbach et al	Engleman et al	Engleman et al	Ropers et al	Ko et al	Marano et al	Schlosser et al	Martuscelli et al	Moore et al	Chiurlia et al	Houslay et al	**Pooled data**
**Year of publication**	1997	1997	2000	2001	2003	2004	2004	2004	2005	2005	2005	
**Method**	Electron beam CT	Spiral CT	Spiral CT	Spiral CT	4 slice CT	4 slice CT	16 slice CT	16 slice CT	4 slice CT	16 slice CT	16 slice CT	
**No. of patients**	25	49	24	65	39	57	51	96	50	52	50	
**Mean age**	64	61	61	67	60	65	65	62	69	63	66	
**Sex (% male)**	92	92	92	82	74	95	76	83%	76	87	86	
**No. grafts**	54	134	78	182	115	122	131	251	150	165	116	**1498**
**IMA**	1	42	20	20	40	95	40	85	38	46	35	**462**
**SVG**	53	92	58	162	54	27	91	166	112	117	77	**1009**
**other**					21					2	4	**27**
**% vein grafts**	98%	69%	74%	89%	47%	22%	69%	66%	66%	70%	66%	
**Mean graft age (yrs)**	6.9	1.8	2.1	7.6	1.2	4.8	5.6	7	8	7.9	7.0	
**Sensitivity**	100%	92%	90.1%	97%	93%	93%	96%	100%	91%	100%	93%	**96%**
**Specificity**	100%	97%	100%	98%	99%	98%	95%	100%	100%	100%	100%	**99%**
**PPV**	100%	99%	100%	97%	93%	93%	81%	100%	100%	100%	100%	**99%**
**NPV**	100%	78%	74%	98%	99%	98%	99%	100%	96%	100%	85%	**95%**
**Accuracy**	100%	93%	92%	98%	98%	97%	95%	100%	97%	100%	95%	**97%**

IMA: internal mammary artery; SVG: saphenous vein graft; PPV: positive predictive value; NPV: negative predictive value.

The main limitation of CT imaging is the high radiation doses that are incurred. This may be of limited relevance in the population under question as it has been shown that the elderly are less susceptible to the lifetime risk of radiation exposure for any given dose[29]. Nonetheless, the radiation dose is double that of invasive coronary angiography. Whilst there are no national dose limits, the doses such as are incurred with CT angiography equate to more than eight times the annual natural background radiation exposure.

Whilst CT angiography has shown promising results in the detection of bypass graft patency, a recent review article concluded that there is a lack of evidence-based data to support its use for evaluation of native vessels in patients presenting with chest pain[30]. CT angiography may be a helpful non-invasive imaging tool in evaluation of bypass grafts on a non-urgent outpatient basis, but clinical trials are needed before it can be used as a tool to evaluate native vessels in patients presenting acutely.

### Study limitations

Image quality is highly dependent upon adequate heart rate control, with a great reduction in image quality in subjects with a heart rate of greater than 70 beats per minute, due to diastolic motion artefacts. While bypass grafts are less susceptible to cardiac motion artefacts than native vessels, this can still be an issue, in particular in posterior vessels, such as grafts to the circumflex artery. Not all of the patients who were recruited were on β-blockade therapy, which meant that image quality was in some cases suboptimal due to diastolic motion artefacts. In order to overcome this major limitation, it would have been helpful to have administered a short acting β-blocker prior to scanning. Additionally, in those patients who had IMA grafts, the increased scan volume necessitated a long breath hold. In some patients, this led to a degree of respiratory artefacts towards the end of the scan time, which tended to coincide with the base of the heart and therefore the distal graft anastamoses.

The use of 16 slice CT scanners has been superseded by newer 64 slice scanners, which have the advantage of narrow collimation and reduced scan times. Meyer *et al *report reliable assessment of graft patency and stenoses in unselected populations using 64 slice scanners[31]. Furthermore, Zhang *et al *highlight improved rated of evaluation of proximal anastamosis, graft, distal anastamosis and run off vessel for 64 slice scanners in comparison to 16 slice CT[32].

## Conclusion

In line with recent studies, we can confirm that MDCT is highly specific for the detection of bypass graft patency. It provides a safe, fast and efficient means of imaging coronary artery bypass grafts but with the disadvantage of a high radiation dose. The introduction of new 64 slice MDCT scanners will result in shorter scan times and may further improve image quality that in turn may enable non-invasive imaging to become the mainstay of assessment of graft patency in the future.

## Competing interests

The author(s) declare that they have no competing interests.

## Authors' contributions

EH carried out the recruitment and running of the study, performed the statistical analysis and drafted the manuscript. TL performed the CT scans, the post-processing and reconstruction of scan data. AS participated in study recruitment and co-ordination of study patients. NU advised regarding data interpretation. GM interpreted and was involved in post-processing of CT scan data and was involved in study design. DN conceived of the study and participated in its design and co-ordination and helped to draft the manuscript. All authors read and approved the final manuscript.
